# Oral Health Status and Treatment Needs Based on Artificial Intelligence (AI) Dental Panoramic Radiograph (DPR) Analysis: A Cross-Sectional Study

**DOI:** 10.3390/jcm13133686

**Published:** 2024-06-25

**Authors:** Natalia Turosz, Kamila Chęcińska, Maciej Chęciński, Iwo Rutański, Marcin Sielski, Maciej Sikora

**Affiliations:** 1 Department of Maxillofacial Surgery, Hospital of the Ministry of Interior, Wojska Polskiego 51, 25-375 Kielce, Poland; marcinsielski@gazeta.pl (M.S.); sikora-maciej@wp.pl (M.S.); 2 Department of Glass Technology and Amorphous Coatings, Faculty of Materials Science and Ceramics, AGH University of Science and Technology, Mickiewicza 30, 30-059 Cracow, Poland; checinska@agh.edu.pl; 3 Department of Oral Surgery, Preventive Medicine Center, Komorowskiego 12, 30-106 Cracow, Poland; 4 Optident sp. z o.o., ul. Eugeniusza Kwiatkowskiego 4, 52-326 Wroclaw, Poland; iworutanski@gmail.com; 5 Department of Biochemistry and Medical Chemistry, Pomeranian Medical University, Powstańców Wielkopolskich 72, 70-111 Szczecin, Poland

**Keywords:** artificial intelligence, dental radiography, panoramic radiography, public health dentistry, DMF Index

## Abstract

**Background:** The application of artificial intelligence (AI) is gaining popularity in modern dentistry. AI has been successfully used to interpret dental panoramic radiographs (DPRs) and quickly screen large groups of patients. This cross-sectional study aimed to perform a population-based assessment of the oral health status and treatment needs of the residents of Kielce, Poland, and the surrounding area based on DPR analysis performed by a high-accuracy AI algorithm trained with over 250,000 radiographs. **Methods:** This study included adults who had a panoramic radiograph performed, regardless of indications. The following diagnoses were used for analysis: (1) dental caries, (2) missing tooth, (3) dental filling, (4) root canal filling, (5) endodontic lesion, (6) implant, (7) implant abutment crown, (8) pontic crown, (9) dental abutment crown, and (10) sound tooth. The study sample included 980 subjects. **Results:** The patients had an average of 15 sound teeth, with the domination of the lower dental arch over the upper one. The most commonly identified pathology was dental caries, which affected 99% of participants. A total of 67% of patients underwent root canal treatment. Every fifth endodontically treated tooth presented a periapical lesion. Of study group members, 82% lost at least one tooth. Pontics were identified more often (9%) than implants (2%) in replacing missing teeth. **Conclusions:** DPR assessment by AI has proven to be an efficient method for population analysis. Despite recent improvements in the oral health status of Polish residents, its level is still unsatisfactory and suggests the need to improve oral health. However, due to some limitations of this study, the results should be interpreted with caution.

## 1. Introduction

### Background

Dental panoramic radiography (DPR), also called orthopantomography, is the most prevalent extraoral technique of dental imaging, enabling the detection of numerous physiological and pathological conditions. It provides a two-dimensional representation of all teeth, the mandible, the maxilla including maxillary sinuses, and temporomandibular joints [1,2]. Many structures imaged simultaneously allow lower radiation doses to detect different disorders. Panoramic radiography is the gold standard in radiological diagnostics. However, it also has limitations. It does not provide detailed information about each tooth but gives an initial oral health assessment. Moreover, a comprehensive analysis is time-consuming and vulnerable to bias due to the varying experiences of the evaluators [3]. High-quality radiographs are essential for accurate human diagnoses and for developing machine learning models that can assist dentists in their practice [4].

Artificial intelligence (AI) has revolutionized healthcare in recent years through early pathology detection and personalized treatments. AI-driven tools are increasingly used in dentistry as they present high performance in detecting and segmenting teeth [5]. Their effectiveness in DPR analysis has seen an upward trend, achieving an accuracy of around 90% [6]. Data-driven AI can assist medical professionals in making time-sensitive decisions [7]. The average time for a dentist to analyze a DPR is over 8 min [8]. For AI models, the exact time depends on the type of software used, and in the case of 2D images, the report is generated up to 10 s [9,10]. Automated methods also eliminate errors associated with clinicians’ mental and eye fatigue, providing superior healthcare quality [5,7]. They can efficiently detect features almost invisible to the human eye. Studies show that AI-based software provides good performance in detecting root canal fillings, crowns, and implants, as well as in predicting prognosis and planning patient-specific treatment [7,11]. This technology can be very useful in population-wide surveillance to perform screening tests, especially in rural communities with a shortage of medical professionals [7]. Despite the great potential of AI applications, their further development and human supervision are still needed [12,13]. Clinicians play a crucial role in ensuring data protection and the ethical use of AI while being able to refine the technology [14].

Screening for oral health needs is commonly performed [15,16]. However, no publications were found where the DMF index score was used to measure a total caries experience in Kielce or the Świętokrzyskie Voivodeship, and no study was identified where AI was used to analyze X-rays and calculate DMF scores. Population screening based on physical examination is expensive and time-consuming. Some pathological changes, such as caries on the proximal surface or periapical lesions, can be difficult to detect only by visual examination. A panoramic radiograph, which supplements a physical exam, could serve as a valuable alternative for gathering information about patients’ oral health.

Artificial intelligence allows the automatic evaluation of DPRs, achieving a high accuracy of about 90% in detecting caries, periodontal bone loss, osteoporosis, maxillary sinusitis, and teeth identification and numbering. The detection of periapical lesions is also characterized by high specificity and sensitivity above 90% [6]. AI algorithms can be used in population-wide surveillance as they perform analyses several times faster than specialists [7,8]. Therefore, it seems reasonable to use AI-based software to perform DPR analyses and assess the oral health status of a larger group of patients.

This cross-sectional study aims to perform a population-based assessment of the oral health status and treatment needs of the inhabitants of Kielce, Poland, and the surrounding area based on an AI-driven DPR analysis. The prevalence and location of decay, dental fillings, root canal fillings, endodontic lesions, implants, implant and dental abutment crowns, pontic crowns, and missing teeth will be investigated.

## 2. Methods

### 2.1. Study Design

This research was designed as a single-arm cross-sectional study following the STROBE Statement: guidelines for reporting observational studies (Strengthening the Reporting of Observational Studies in Epidemiology)and the principles of the Declaration of Helsinki and was approved by the Bioethics Committee in Kielce at the Świętokrzyska Chamber of Physicians (approval number: 2.3/2023). The study protocol was developed based on the STROBE checklist. Characteristics of the study design are presented in Table 1 [17].

### 2.2. Setting

**Figure 1:**
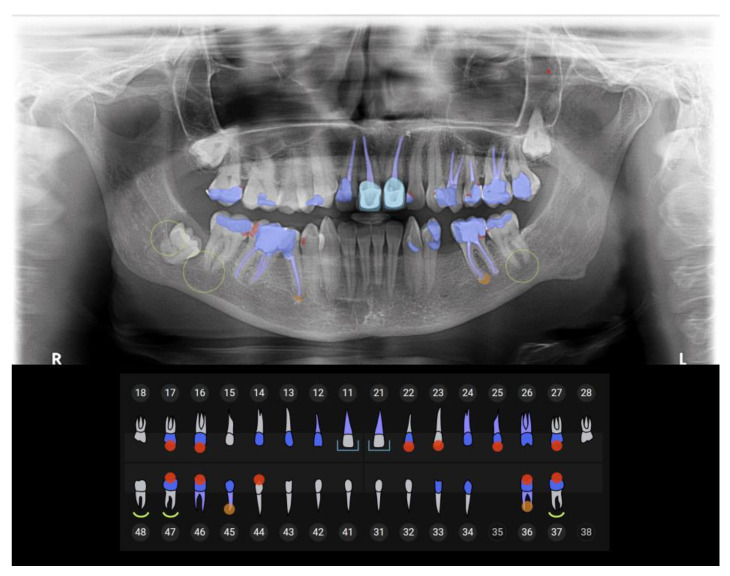
The view of DPR analysis performed by AI.

The patients included in this study were admitted between September 2022 and June 2023 to the radiology department located in Kielce, a city in southern Poland. The department is located near communication hubs serving public transport within Kielce County, a unit of territorial administration that includes the city of Kielce and surrounding villages, with approximately 207,000 inhabitants. The radiology department performs both insurance-covered and commercial medical procedures. This allows for a versatile range of services that meet the diverse needs of all patients. High-resolution panoramic radiographs were taken using the device Carestream CS9600 with adjustable exposure conditions set to 60–90 kV and 2–15 mA. Then, AI Insights software (version CSI8 server ver. 3.12; Carestream Health, Rochester, NY, USA) analyzed panoramic X-ray images in June 2023 after anonymizing the data. The algorithm is integrated with CS Imaging v8 software, thanks to which we could quickly retrieve an automated dental chart for each DPR we had. The algorithm was trained with over 250,000 panoramic radiographs previously described by professional radiologists. The proven accuracy of AI in image classification is 99%, and the accuracy in detecting periapical lesions on panoramic radiographs is up to 95% [18,19]. AI Insights assesses the digital image with one click in seconds, displaying the findings and highlighting them in color directly on the image (Figure 1). The user can modify the description, e.g., by selecting caries that the program did not recognize. It is also possible to generate a report in PDF format with basic information about the patient and radiation doses used.

### 2.3. Participants

The eligibility criteria are presented in Table 2. All patients had an X-ray taken on the day of admission to the radiology department. Only DPRs that met appropriate quality standards, such as clearly visible teeth and outlines of the jawbone, horizontal or slightly raised upwards occlusal plane, were qualified for analysis. DPRs with artifacts and positioning imperfections were excluded.

### 2.4. Variables

The variables presented in Table 3 were used for analysis, taking into account their positions. To describe the results of this study, two dental notation systems were applied: FDI World Dental Federation (FDI) notation and the Universal Numbering System (UNS), also called the “American system”. The adopted methodology classified teeth with pathologies not listed in Table 3 as sound, e.g., teeth with dental developmental anomalies or marginal periodontal loss.

### 2.5. Data Sources/Measurement

The source of the data was a series of panoramic X-ray images taken using Carestream CS9600. For the automatic analysis, we used a dedicated AI algorithm, available since 2022, which was trained with over 250,000 DPRs analyzed by medical professionals to detect dental caries, endodontic lesions, fillings, different types of prosthetic restorations, and implants.

### 2.6. Bias

The sample was selected from consecutive patients, which resulted in a random pattern of values of the evaluated variables. An evaluation in an AI-driven program was performed by a single investigator and was always conducted in the same mode. Apart from sampling, there was no other risk of bias in the AI evaluation.

### 2.7. Study Size

According to the WHO sample size calculator for a 1.96 level of confidence (a 95% confidence interval), 0.05 margin of error, unknown baseline levels of indicators, simple random sample (design effect = 1), and lack of subgroups, the appropriate sample size should be 384.16 subjects. We determined a sample size of 1025 participants due to the research budget.

### 2.8. Quantitative Variables

The values of the variables (1) dental caries, (2) missing tooth, (3) dental filling, (4) root canal filling, (5) endodontic lesion, (6) implant, (7) implant abutment crown, (8) pontic crown, (9) dental abutment crown, and (10) sound tooth were grouped depending on the tooth number, according to the FDI World Dental Federation notation and the Universal Numbering System.

### 2.9. Statistical Methods

The acquired data were assessed in the Excel program (Microsoft Corporation, Redmond, WA, USA). Point prevalence was used to measure the frequency of studied variables in the randomly selected sample from the population of Kielce County. It is the proportion of subjects that have the characteristic at a given moment in time [21,22]. The Pearson correlation coefficients, which give the strength of the linear relationship between two variables, were also calculated and presented in a correlation matrix. The formula value lies between −1 and 1, which correspond to perfect negative and perfect positive linear relationships, respectively. If the value is zero, then the variables have no correlation [23].

## 3. Results

### 3.1. Participants

**Figure 2:**
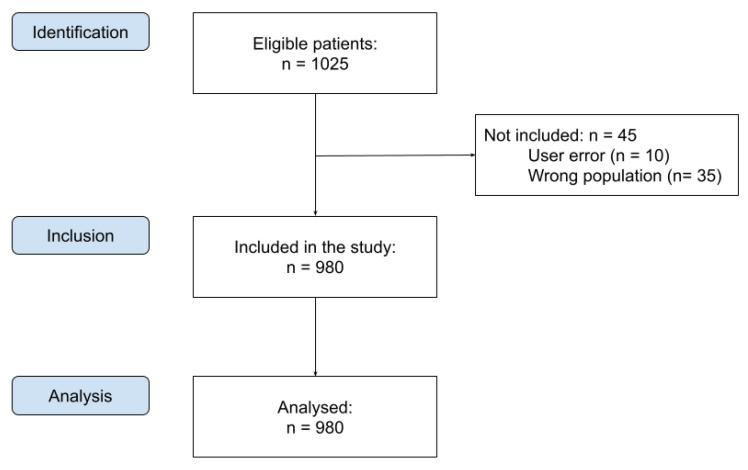
Flowchart of patient selection.

This study involved 1025 patients. The radiographs were analyzed by AI Insights software (version CSI8 server ver. 3.12; Carestream Health, Rochester, NY, USA), resulting in 980 correctly performed analyses. Data on 45 patients were not obtained due to user error while using the AI program (n = 10) or them not meeting the inclusion criteria (n = 35) because the DPRs were analyzed with mixed dentition (Figure 2).

### 3.2. Descriptive Data

**Figure 3:**
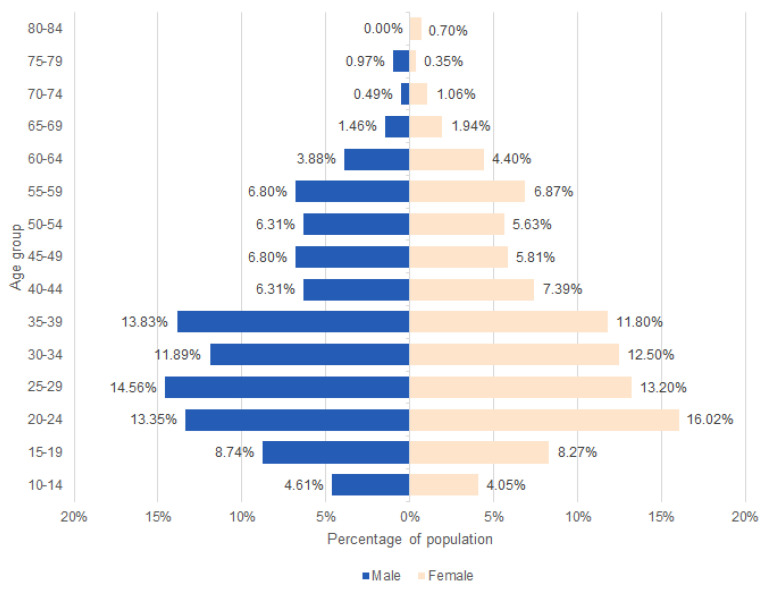
The age structure of the included participants.

For DPR analysis, 980 patients (568 women and 412 men) were included in this study. Figure 3 presents the age structure of participants. The male-to-female ratio was 0.73. The average age of patients was 35.6 (SD = 15.0; median = 33). The oldest in the study sample was a man of 77 years and a woman of 81 years. The patients were grouped into 15 age ranges for demographic assessment, with 20–24, 25–29, 30–34, and 35–39 predominating. The loss of all deciduous dentition determined the lower age limit. The youngest patient in both the male and female groups was 11 years old.

### 3.3. Outcome Data

Table 4 and Table 5 present the results of the DPR analysis of the 980 patients. Of all the 32 teeth positions in the 980 patients (31,360 items), the most common diagnosis was a sound tooth. In the study population, sound teeth were identified 16% more often in the lower arch than in the upper arch. The upper arch was more likely to have caries, dental fillings, and root canal fillings than the lower arch, by 18%, 12%, and 8%, respectively. Periapical lesions in both arches occurred at similar levels. Tooth 16 was most often affected by dental caries and filled. First molars were also most frequently treated endodontically and had periapical lesions. Prosthetic restorations were far more common in the upper arch, with crowns almost four times more frequent than pontics. Implants occurred rarely and were located mainly in the upper arch. Patients had an average of five missing teeth.

### 3.4. Main Results

**Figure 4:**
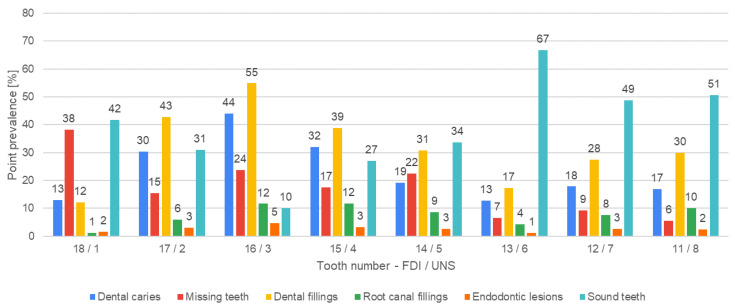
Distribution of decayed, missing, and filled teeth, root canal fillings, endodontic lesions, and sound teeth according to teeth positions in the upper-right quadrant.

**Figure 5:**
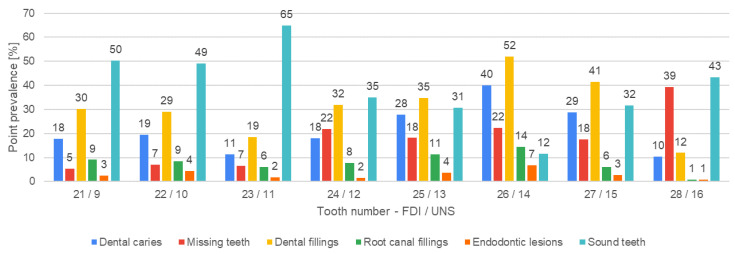
Distribution of decayed, missing, and filled teeth, root canal fillings, endodontic lesions, and sound teeth according to teeth positions in the upper-left quadrant.

**Figure 6:**
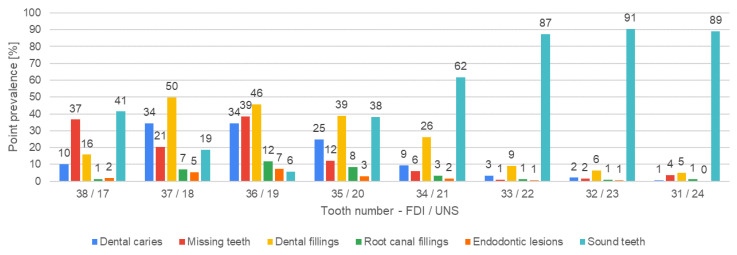
Distribution of decayed, missing, and filled teeth, root canal fillings, endodontic lesions, and sound teeth according to teeth positions in the lower-right quadrant.

**Figure 7:**
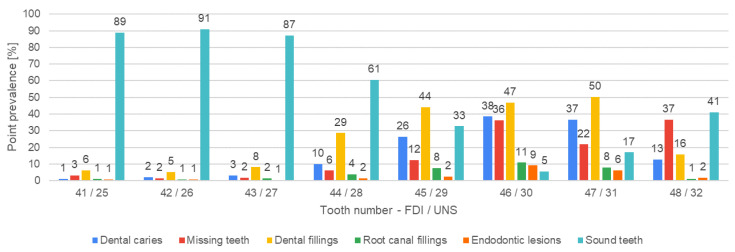
Distribution of decayed, missing, and filled teeth, root canal fillings, endodontic lesions, and sound teeth according to teeth positions in the lower-left quadrant.

**Figure 8:**
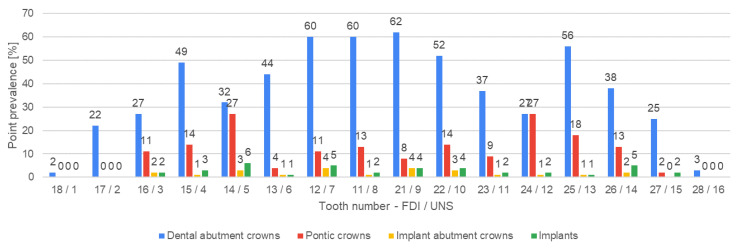
Distribution of dental and implant abutment crowns, pontic crowns, and implants according to teeth positions in the upper arch.

**Figure 9:**
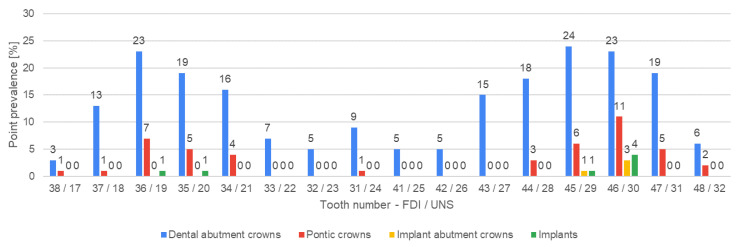
Distribution of dental and implant abutment crowns, pontic crowns, and implants according to teeth positions in the lower arch.

Figure 4, Figure 5, Figure 6, Figure 7, Figure 8 and Figure 9 present the distribution of (1) decayed, (2) missing, and (3) filled teeth, (4) root canal fillings, (5) endodontic lesions, (6) sound teeth, (7) dental, and (8) implant abutment crowns, (9) pontic crowns, and (10) implants in the upper and lower arch. The values of point prevalence for consecutive teeth do not add up to 100% because a given tooth may have several diagnoses. The most common sound tooth in the maxillary arch was 13/6, and in the mandibular arch, teeth in the anterior segment 33/22–43/27 usually did not present any pathological conditions. Tooth 16/3 was the most frequently affected by caries and filled. The most common missing teeth were third molars. Periapical lesions occurred most often in the first molars. Dental abutment crowns mostly restored upper incisors and first premolars.

### 3.5. Subgroup Analyses

**Figure 10:**
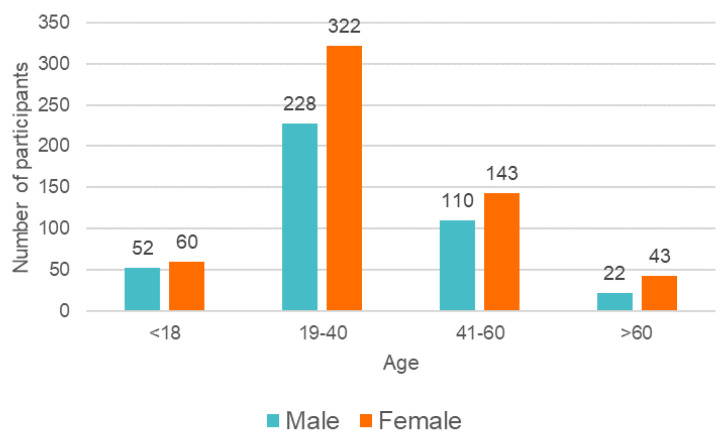
The age structure of participants in consecutive groups.

The analysis was also performed in four age groups: up to 18 years old, 19–40, 41–60, and over 60 years old (Figure 10). Table 6, Table 7, Table 8 and Table 9 present the analysis results in consecutive age groups, considering the most common and rarest teeth with a given finding.

### 3.6. Other Analyses

An analysis of the relationship between the presence of periapical lesions and root canal treatment was also performed. The examined material included 1918 (7%) endodontically treated teeth out of all 26,294 teeth identified as present. In the group of teeth after or during root canal treatment, there were 360 (19%) cases of periapical radiological radiolucency. The correlation coefficient between endodontic treatment and the presence of periapical radiolucency was 0.23. Among the teeth with identified periapical radiolucency, 552 (59%) of 882 had no evidence of endodontic treatment.

Table 10 presents the correlation matrix of the analyzed variables. A high positive correlation (>0.7) occurred between the missing teeth and age and between the presence of implants and implant abutment crowns. In this study, 61% of the implants had dental crowns attached at the time of exposure. A moderate positive correlation (0.5–0.7) was found between pontic crowns and dental abutment crowns.

## 4. Discussion

### 4.1. AI Software

The use of AI algorithm optimized the work of clinicians and validated their evaluation of DPRs. According to our calculations, the average time for an equally detailed analysis performed by a dentist is approximately 4 min, while the algorithm performs it in seconds. This shows that using new technologies can boost practice performance. The integration of algorithm into the existing workflow is seamless. Color-coded findings facilitate image reviews and communication with patients who better understand their treatment needs. Consequently, modern AI tools help build trust between practitioners and patients. Thanks to additional analyses, especially inexperienced dentists feel more confident, reducing undiagnosed cases and making better clinical decisions [24].

### 4.2. Dental Caries

Dental caries, the most prevalent disease worldwide, was detected in 973 out of 980 included patients (99%). No carious lesions were detected in only seven participants of this study. According to the latest official report from 2021, the prevalence of caries in Poland is almost 100% in the adult population [16]. A significant relationship was found between the dental caries and gender [16]. In our study, the largest number of decayed teeth (19 teeth) was detected in a 35-year-old woman. The most common caries location was tooth FDI 16/UNS 3 (432 teeth) and the rarest was FDI 31/UNS 24 (7 teeth). These teeth were also filled the most frequently and least frequently, respectively. Rarely, caries appeared in the lower front teeth, FDI 34–44/UNS 21–28 (less than 100 teeth). Tooth FDI 42/UNS 26 was the most common healthy tooth, with no pathology or restoration present. This result coincides with other studies showing that the maxillary and mandibular molars are the most susceptible to caries, while the mandibular central incisors are the least susceptible [25,26]. Hassan et al. revealed the mesial surface of the maxillary permanent first molar is more prone to dental caries than the distal one [27]. Caries is also more prevalent in the upper arch than in the lower arch, which was confirmed in this research: 59% of dental caries lesions occurred in the upper arch and 41% in the lower arch [25].

### 4.3. Missing Teeth

In the research material, the most frequently missing teeth were (1) FDI 28/UNS 16 (385 times), (2) FDI 36/UNS 19 (379 times), (3) FDI 18/UNS 1 (375 times), (4) FDI 48/UNS 32 (360 times), and (5) FDI 38/UNS 17 (359 times). The least frequently missing tooth was FDI 33/UNS 22 (8 times). There are various causes of tooth loss, such as dental caries, periodontal disease, trauma, failed endodontic treatment, incorrect position, or tooth agenesis (its congenital absence) [28,29]. In the study of Scheiwiller et al., the prevalence of 50.8% for third-molar agenesis occurred in the group of patients with agenesis of teeth other than the third molar, which suggests that third molars are more vulnerable to genetic factors associated with tooth agenesis. An evolutionary trend toward reduced molar number is probable [29]. Some orthodontists and oral surgeons recommend extracting third molars to prevent the crowding of teeth upon their eruption [30]. However, recent studies do not present sufficient evidence to advocate the preventive removal of wisdom teeth to obtain occlusal stability [31,32]. The study of Dosumu et al. showed poor knowledge of the consequences of missing teeth among patients with partial edentulism [28]. In the study, 177 patients (18%) did not lose any teeth, and 803 patients (82%) had at least one lost tooth. Only one patient was edentulous. According to a study from 2021, the percentage of Polish people who have at least 20 teeth, preserving chewing function, has increased over recent years. In 2017, 97% of patients aged 35–44 had 20 teeth, while in our study, this percentage was similar at 94.8% [16].

### 4.4. Endodontic Lesions and Treatment

In this study, 656 patients (66.9%) underwent root canal treatment (RCT). The most frequently endodontically treated teeth were FDI 26/UNS 14 (141 times) and FDI 36/UNS 19 (118 times). The least frequent teeth with filling in the root canals were FDI 28, 42/UNS 16, 26 (8 times). According to the systematic review of León-López et al., considering the prevalence of RCT treatment worldwide, more than half of the studied population has at least one root-filled tooth [33]. Inflammation of the periapical periodontium, called apical periodontitis, occurs due to untreated irreversible pulpitis and pulp necrosis. It is commonly accompanied by periapical bone resorption [34]. In this study, periapical lesions were most frequently located at tooth FDI 46/UNS 30 (89 times) and least frequently at tooth FDI 31/UNS 24 (3 times). They can also be the effect of RCT performed incorrectly. In the study of Özbaş et al. on a Turkish subpopulation, 40% of endodontically treated teeth had periapical lesions, which indicated the necessity of improving the technical quality of root canal filling by dentists [35]. According to a report by Alnowailaty et al., most identified untreated canals occurred in maxillary and mandibular first molars, resulting in apical periodontitis [36]. In our study, every fifth endodontically treated tooth had a periapical lesion (19%). Some teeth could be treated properly, but the lesion had not yet healed. It has been reported that 50% of cases exhibit signs of healing after 6 months, whereas after 12 months, 88% of these lesions are completely healed [37]. Moreover, a longer healing process occurs in older patients and when the area of the bone loss is more advanced. Sometimes, the treatment observation period is up to 18 months [38].

Almost 60% of periapical radiolucencies, known as “endodontic lesions”, were identified in teeth without evidence of root canal treatment. Despite the similar radiological picture, this group of diagnoses requires differentiation by physical examination. These may include, among others, true periapical inflammatory lesions, root tips during natural development, bone dysplasia, natural anatomical structures (mainly mental foramina), natural arrangement of bone trabeculae imitating pathology, and radiological imaging of the consequences of orthodontic tooth displacement and tumors [39,40,41].

### 4.5. Restoring Missing Teeth with Dental Implants and Bridges

In the analyzed material, implants appeared relatively rarely (2% of participants). According to a recent study from 2023, Polish patients show limited knowledge of dental implants [42]. Their major concern about this treatment option is the high cost and the need for surgery. [42] Implants were often inserted around tooth FDI 26/UNS 14 (6 times). In the study group, implants were not placed in the place of teeth FDI 18, 17, 28, 38, 37, 34–44, 47, 48/UNS 1, 2, 16, 17, 18, 21–28, 31, 32. According to a report concerning trends in dental implants in the US in 1999–2016, most were placed in posterior sites, almost equally in the maxilla and mandible [43]. The anterior maxillary region, being an aesthetic zone, requires special attention in the treatment plan to eliminate the risk of positioning errors, considering gingival phenotype, the width of the edentulous space, and bone anatomy at the alveolar crest [44]. A detailed assessment of the distribution of dental implants based on the study material may not be reliable, as only 2% of participants (23 people) underwent this treatment method. Bridge pontics were present more often to replace missing teeth (8.78%). This cross-sectional study shows current oral statuses. Therefore, it cannot be concluded that patients prefer bridges over implants, as many restorations were placed when the latter were less available.

### 4.6. Dental Crowns

The most common restored tooth with a crown was FDI 21/UNS 9 (62 times). The anterior region is an important area for oral aesthetics; therefore, it requires high-quality reconstruction. Chairside restoration is not always possible due to the significant destruction of the hard tissues of the tooth. In this study, 21.4% of teeth with root canal fillings were restored with a crown. Tikku et al. show that the coronal coverage significantly improves the success rate of endodontic treatment [45].

### 4.7. Limitations

Caries lesions confined to the enamel may not be visible on radiographs until the demineralization of the tooth structure is approximately 30–60% [46]. For this reason, incipient lesions can be difficult to detect not only by the dentist but also by the AI algorithm. Moreover, despite the significant diagnostic value of DPRs, intraoral bitewing radiography is superior to panoramic radiography in detecting proximal caries of premolars and molars [47]. The sensitivity of caries detection in DPRs is about 60% [48], which means that there may be more analyzed teeth with caries. Sometimes dental crowding can also make it difficult to detect caries, especially in the incisor region, where superimposition of the cervical spine appears as an anatomical ghost shadow [49].

Panoramic radiography has some disadvantages. It provides less accurate information about dental diseases than intraoral radiographs. Imaging errors such as significantly overlapped structures, shadows of soft tissues or anatomical air spaces, and distortion may often be seen [50]. Such low-quality images may decrease algorithm performance if they are used in building machine learning models [4].

In Poland, DPRs can be taken only in patients with indications confirmed by a written referral from the dentist or physician. This is due to legal regulations regarding radiological protection [51]. Therefore, radiographs do not exist for patients without any suspected pathologies.

Another limitation is the assessment of only hard tissue pathologies detectable on radiographs. It should be emphasized that analyzing a DPR will not replace a medical interview and physical examination. Nevertheless, we believe that a cross-sectional study based on DPRs assessed by AI, conducted on a large sample, provides basic knowledge about the dental needs of the population and will help to plan further, more detailed research and preventive programs.

In this study, we used an algorithm, which does not have an accuracy of 100%; therefore, some diagnoses may have been incorrect. However, according to a recent systematic review, AI models achieve an accuracy above 90% in detecting caries and teeth identification and numbering [6]. Detecting periapical lesions is characterized by high sensitivity (99.75%) and specificity (92%) as well [6]. AI models can also be effectively used in periodontics, providing accuracy above 81% in detecting periodontal bone loss [52,53]. Very high accuracy, between 94 and 98%, also occurred in implant type recognition [54].

AI models appear to be powerful diagnostic tools, as the DPR analyses performed by AI models are similar to those made by humans. Although the difference seems subtle, it should be taken into account, and its value should be updated with technological progress. Therefore, there is a pressing need for current clinical research on this topic.

## 5. Conclusions

Despite improvement in the oral health of Polish people observed in recent years, its level is still unsatisfactory. Our automatic analysis of 980 DPRs of patients with permanent teeth aged 11–81 years showed that dental caries occurred in almost all the participants. The findings also suggest the vital role of preventive oral healthcare programs, developing new oral health policies, allocating dedicated funds for oral health at the Ministry of Health, and increasing access to affordable essential oral health care. AI-driven tools can be very useful in quickly screening a large group of patients and addressing their needs. Early detection and identification of pathologic conditions are key for timely treatment. By incorporating AI software as a second opinion, dentists can reduce untreated cases, offering enhanced protection for patients. However, due to some limitations of this study, the results should be interpreted with caution.

## Figures and Tables

**Table 1:** Study design.

Study Design Feature	Applied Study Design
Direction of data collection	Retrospective
Number of gates (sets of eligibility criteria)	Double gate (AI, human)
Participant sampling method	Consecutive
Method of allocating participants to index tests	Each participant received all index tests
Number of reference standards	Single test standard
Limited verification	Full verification (not limited)

**Table 2:** Inclusion and exclusion criteria.

Domain	Criteria for Inclusion	Criteria for Exclusion
Indications	Typical indications for DPR imaging confirmed by a written referral from the dentist or physician (both screening tests and tests performed for treatment purposes were allowed)	Not applicable
Dentition	Not applicable	Patients with mixed or primary dentition
Age	Patients of any age	No age restrictions applied due to the limitation in the dentition category
Sex	All genders	No gender restrictions
Quality of DPRs	Correctly performed DPR in accordance with the criteria of the Polish Ministry of Health [20]	AI error resulting in no results or partial results

**Table 3:** Variables.

Abbreviation	Name of the Variable	Description
D	Dental caries	Presence of at least one cavity (carious or non-carious) in a given tooth
M	Missing tooth	Absence of any tooth remnants in a given location
F	Dental filling	Presence of at least one filling in a given tooth
R	Root canal filling	Presence of at least one filled root canal (completely or partially)
E	Endodontic lesion	Periapical radiological radiolucency primarily suggesting periapical inflammation
I	Implant	Radiological shading in the shape of an intraosseous dental implant
A	Implant abutment crown	Prosthetic crown based on an implant
P	Pontic crown	Prosthetic bridge span (prosthetic crown without direct support)
C	Dental abutment crown	A prosthetic crown supported on a tooth
S	Sound tooth	A tooth without signs of the above-mentioned pathologies or signs of the above-mentioned treatment methods

**Table 4:** Total number of findings, average findings per patient, and number of findings in the upper and lower arch in the included participants of this study.

Finding	Total Number	Average per Patient	Standard Deviation	Upper Arch	Lower Arch
Sound tooth	14,533	14.83	6.99	6141	8392
Dental filling	8882	9.06	4.80	4942	3940
Dental caries	5975	6.10	3.11	3526	2449
Missing tooth	5066	5.17	5.68	2714	2352
Root canal filling	1918	1.96	2.18	1227	691
Endodontic lesion	882	0.90	1.19	447	435
Dental abutment crown	806	0.82	1.76	596	210
Pontic crown	217	0.22	0.89	171	46
Implant	46	0.05	0.38	39	7
Implant abutment crown	28	0.03	0.32	24	4

**Table 5:** Maximum and minimum number of occurrences of each finding in the included participants of this study.

	The Most Common Tooth (Frequency) FDI/UNS	The Least Common Tooth (Frequency) FDI/UNS
Dental caries	16/3 (432 times)	31/24 (7 times)
Missing teeth	28/16 (385 times)	33/22 (8 times)
Dental filling	16/3 (538 times)	31/24 (51 times)
Root canal filling	26/14 (141 times)	28/16, 42/26 (8 times)
Endodontic lesion	46/30 (89 times)	31/24 (3 times)
Dental abutment crown	21/9 (62 times)	18/1 (2 times)
Pontic abutment crown	14/5, 24/12 (27 times)	18/1, 17/2, 28/16, 33/22, 32/2, 41/25–44/27 (0 times)
Implant abutment crown	12/7, 21/9 (4 times)	18/1, 17/2, 27/15–44/28, 47/31, 48/32 (0 times)
Implant	14/5 (6 times)	18/1, 17/2, 28/16–37/18, 34/21–44/28, 47/31, 48/32 (0 times)
Sound tooth	42/26 (892 times)	46/30 (53 times)

**Table 6:** Characteristics of participants under 18 years old.

	The Most Common Tooth (Frequency) FDI/UNS	The Least Common Tooth (Frequency) FDI/UNS	Average per Patient
Dental caries	36/19 (57 times)	13/6, 38/17, 33/22–43/27, 48/32 (0 times)	4.01
Missing teeth	18/1, 28/16, 48/32 (11 times)	15/4, 13/6, 11/8–22/10, 37/18, 33/22–43/27 (0 times)	0.79
Dental filling	16/3, 36/19 (60 times)	18/1, 28/16, 38/17 (0 times)	4.5
Root canal filling	46/30 (6 times)	18/1, 17/2, 14/5, 13/6, 11/8, 22/10, 23/11, 25/13, 28/16, 38/17, 35/20–44/28, 47/31, 48/32 (0 times)	0.27
Endodontic lesion	46/30 (7 times)	18/1, 17/2, 13/6, 24/12, 27/15, 28/16, 33/22–43/27, 48/32 (0 times)	0.44
Dental abutment crown	16/3, 26/14 (2 times)	18/1, 17/2, 14/5, 13/6, 11/8, 22/10, 23/11, 25/13, 27/15–37/18, 35/20–45/29, 47/31, 48/32 (0 times)	0.09
Pontic abutment crown	1 (1 time)	18/1–12/7, 21/9–48/32 (0 times)	0.01
Implant abutment crown	0	0	0
Implant	0	0	0
Sound tooth	13/6, 42/26 (110 times)	36/19 (27 times)	24.5

**Table 7:** Characteristics of participants between 19 and 40 years old.

	The Most Common Tooth (Frequency) FDI/UNS	The Least Common Tooth (Frequency) FDI/UNS	Average per Patient
Dental caries	16/3 (270 times)	41/25 (2 times)	6.6
Missing teeth	28/16, 36/19 (157 times)	42/26 (1 time)	3.2
Dental filling	16/3 (352 times)	32/23, 42/26 (19 times)	9.9
Root canal filling	36/19 (83 times)	33/22 (0 times)	1.8
Endodontic lesion	46/30 (58 times)	43/27 (0 times)	0.9
Dental abutment crown	25/13 (26 times)	38/17, 33/22, 41/25, 42/26, 48/32 (0 times)	0.4
Pontic abutment crown	24/12 (9 times)	18/1, 17/2, 13/6, 27/15–35/20, 33/22–48/32 (0 times)	0.1
Implant abutment crown	21/9 (2 times)	18/1–15/4, 13/6, 11/8, 22/10–44/28, 47/31, 48/32 (0 times)	0
Implant	14/5 (3 times)	18/1–15/4, 13/6, 11/8, 22/10, 23/11, 25/13, 27/15–37,18, 35/20–44/28, 47/31, 48/32 (0 times)	0
Sound tooth	42/26 (525 times)	46/30 (24 times)	16

**Table 8:** Characteristics of participants between 41 and 60 years old.

	The Most Common Tooth (Frequency) FDI/UNS	The Least Common Tooth (Frequency)	Average per Patient
Dental caries	17/2, 16/3 (91 times)	31/24 (2 times)	6.2
Missing teeth	36/19 (163 times)	33/22 (3 times)	8.9
Dental filling	17/2 (131 times)	31/24 (15 times)	9.9
Root canal filling	26/14 (44 times)	32/23, 41/25, 48/32 (3 times)	2.9
Endodontic lesion	46/30 (23 times)	31/24, 48/32 (2 times)	1
Dental abutment crown	12/7 (31 times)	18/1, 28/16 (1 time)	1.7
Pontic abutment crown	14/5 (17 times)	18/1, 17/2, 28/16, 33/22, 32/23, 41/25–43/27 (0 times)	0.5
Implant abutment crown	14/5, 12/7, 21/9, 22/10, 46/30 (2 times)	18/1, 17/2, 13/6, 23/11, 27/15–45/29, 47/31, 48/32 (0 times)	0.1
Implant	15/4, 14/5, 12/7, 22/10, 26/14, 46/30 (3 times)	18/1, 17/2, 13/6, 28/16–36/19, 34/21–45/29, 47/31, 48/32 (0 times)	0.1
Sound tooth	42/26 (216 times)	46/30 (0 times)	9.6

**Table 9:** Characteristics of participants over 60 years old.

	The Most Common Tooth (Frequency) FDI/UNS	The Least Common Tooth (Frequency)	Average per Patient
Dental caries	13/6 (21 times)	38/17 (0 times)	4.9
Missing teeth	38/17 (58 times)	33/22 (2 times)	15
Dental filling	45/29 (26 times)	38/17 (3 times)	6.5
Root canal filling	35/20 (14 times)	28/16, 38/17 (0 times)	2.4
Endodontic lesion	25/13, 37/18, 47/31(5 times)	13/6, 11/8, 38/17, 32/23, 31/24 (0 times)	0.8
Dental abutment crown	15/4, 12/7 (13 times)	18/1, 28/16, 38/17, 32/23, 41/25, 42/26 (0 times)	2.4
Pontic abutment crown	24/12 (6 times)	18/1, 17/2, 28/16–37/18, 35/20, 33/22–43/27, 48/32 (0 times)	0.7
Implant abutment crown	16/3, 13/6, 12/7, 22/10, 23/11, 26/14 (1 time)	18/1, 17/2, 15/4, 14/5, 11/8, 21/9, 24/12, 25/13, 27/15–48/32 (0 times)	0.1
Implant	16/3, 13/6, 12/7, 22/10, 23/11, 26/14 (1 time)	18/1, 17/2, 15/4, 14/5, 11/8, 21/9, 24/12, 25/13, 27/15–48/32 (0 times)	0.1
Sound tooth	32/23, 43/27 (42 times)	37/18, 36/19 (0 times)	6.6

**Table 10:** A correlation matrix. Correlation coefficients with absolute values greater than or equal to 0.5 are marked with an asterisk.

	Age	D	M	F	R	E	I	A	P	C
**Age**										
**D**	0.01									
**M**	0.71 *	−0.12								
**F**	0.09	0.38	−0.21							
**R**	0.35	0.27	0.15	0.39						
**E**	0.12	0.37	0.08	0.01	0.23					
**I**	0.12	−0.06	0.06	−0.06	0.12	−0.06				
**A**	0.10	−0.08	0.05	−0.06	0.10	−0.05	0.88 *			
**P**	0.29	−0.04	0.10	−0.10	0.19	0.01	0.27	0.32		
**C**	0.44	0.02	0.23	−0.04	0.44	0.08	0.28	0.26	0.63 *	

D—dental caries, M—missing teeth, F—dental filling, R—root canal filling, E—endodontic lesion, I—implant, A—implant abutment crown, P—pontic crown, C—dental abutment crown.

## Data Availability

The raw data supporting the conclusions of this article will be made available by the authors on request.

## References

[1] Różyło-Kalinowska I. (2021). Panoramic Radiography in Dentistry. Clin. Dent. Rev..

[2] Iannucci J., Howerton L.J. (2021). Dental Radiography—Principles and Techniques.

[3] Lee J.-H., Han S.-S., Kim Y.H., Lee C., Kim I. (2020). Application of a Fully Deep Convolutional Neural Network to the Automation of Tooth Segmentation on Panoramic Radiographs. Oral Surg. Oral Med. Oral Pathol. Oral Radiol..

[4] Delamare E., Fu X., Huang Z., Kim J. (2024). Panoramic Imaging Errors in Machine Learning Model Development: A Systematic Review. Dentomaxillofacial Radiol..

[5] Leite A.F., Gerven A.V., Willems H., Beznik T., Lahoud P., Gaêta-Araujo H., Vranckx M., Jacobs R. (2021). Artificial Intelligence-Driven Novel Tool for Tooth Detection and Segmentation on Panoramic Radiographs. Clin. Oral Investig..

[6] Turosz N., Chęcińska K., Chęciński M., Brzozowska A., Nowak Z., Sikora M. (2023). Applications of Artificial Intelligence in the Analysis of Dental Panoramic Radiographs: An Overview of Systematic Reviews. Dentomaxillofacial Radiol..

[7] Patil S., Albogami S., Hosmani J., Mujoo S., Kamil M.A., Mansour M.A., Abdul H.N., Bhandi S., Ahmed S.S.S.J. (2022). Artificial Intelligence in the Diagnosis of Oral Diseases: Applications and Pitfalls. Diagnostics.

[8] Zadrożny Ł., Regulski P., Brus-Sawczuk K., Czajkowska M., Parkanyi L., Ganz S., Mijiritsky E. (2022). Artificial Intelligence Application in Assessment of Panoramic Radiographs. Diagnostics.

[9] The Digital Landscape of Dentistry: Dental AI Software—Diagnocat. https://diagnocat.com/eu/blog/the-digital-landscape-of-dentistry-dental-ai-software/.

[10] PRESS RELEASE: Artificial Intelligence Takes Root—AI Insights Delivers Automatic Panoramic Image Analysis and Reporting in Seconds. https://www.carestreamdental.com/en-emea/training-resources/emea-newsroom/english/posts/2022/press-release-artificial-intelligence-takes-root--ai-insights-delivers-automatic-panoramic-image-analysis-and-reporting-in-seconds/.

[11] Bonfanti-Gris M., Garcia-Cañas A., Alonso-Calvo R., Salido Rodriguez-Manzaneque M.P., Pradies Ramiro G. (2022). Evaluation of an Artificial Intelligence Web-Based Software to Detect and Classify Dental Structures and Treatments in Panoramic Radiographs. J. Dent..

[12] Putra R.H., Doi C., Yoda N., Astuti E.R., Sasaki K. (2022). Current Applications and Development of Artificial Intelligence for Digital Dental Radiography. Dento Maxillo Facial Radiol..

[13] Pesapane F., Codari M., Sardanelli F. (2018). Artificial Intelligence in Medical Imaging: Threat or Opportunity? Radiologists Again at the Forefront of Innovation in Medicine. Eur. Radiol. Exp..

[14] Shafi I., Fatima A., Afzal H., Díez I.d.l.T., Lipari V., Breñosa J., Ashraf I. (2023). A Comprehensive Review of Recent Advances in Artificial Intelligence for Dentistry E-Health. Diagnostics.

[15] Chan A.K.Y., Tamrakar M., Jiang C.M., Lo E.C.M., Leung K.C.M., Chu C.H. (2021). A Systematic Review on Caries Status of Older Adults. Int. J. Environ. Res. Public. Health.

[16] Olczak-Kowalczyk D. Monitorowanie Stanu Zdrowia Populacji Polskiej w Latach 2016–2020. Choroba Próchnicowa i Stan Tkanek Przyzębia Populacji Polskiej. Podsumowanie Wyników Badań z Lat 2016–2019. Sekcja Druków Uczelnianych Warszawskiego Uniwersytetu Medycznego Warszawa, Poland, 2021. https://www.gov.pl/attachment/e837445b-41ef-4b49-b261-d21d869e0018/.

[17] Yang B., Olsen M., Vali Y., Langendam M.W., Takwoingi Y., Hyde C.J., Bossuyt P.M.M., Leeflang M.M.G. (2021). Study Designs for Comparative Diagnostic Test Accuracy: A Methodological Review and Classification Scheme. J. Clin. Epidemiol..

[18] Cejudo J.E., Chaurasia A., Feldberg B., Krois J., Schwendicke F. (2021). Classification of Dental Radiographs Using Deep Learning. J. Clin. Med..

[19] Ekert T., Krois J., Meinhold L., Elhennawy K., Emara R., Golla T., Schwendicke F. (2019). Deep Learning for the Radiographic Detection of Apical Lesions. J. Endod..

[20] (2015). Dziennik Urzędowy Ministra Zdrowia Obwieszczenie Ministra Zdrowia z Dnia 10 Listopada 2015 r. w Sprawie Ogłoszenia Wykazu Wzorcowych Procedur Radiologicznych z Zakresu Radiologii—Diagnostyki Obrazowej i Radiologii Zabiegowej. https://dziennikmz.mz.gov.pl/DUM_MZ/2015/78/akt.pdf.

[21] González-Ramírez A.R., Rivas-Ruiz F. (2010). Measures of Frequency, Magnitude of Association and Impact in Epidemiology. Allergol. Immunopathol..

[22] What Is Prevalence?—National Institute of Mental Health (NIMH). https://www.nimh.nih.gov/health/statistics/what-is-prevalence.

[23] Correlation Coefficient—An Overview|ScienceDirect Topics. https://www.sciencedirect.com/topics/earth-and-planetary-sciences/correlation-coefficient.

[24] Carestream Dental|AI Insights. https://www.carestreamdental.com/en-gb/csd-products/software/imaging-software/ai-insights/.

[25] Demirci M., Tuncer S., Yuceokur A.A. (2010). Prevalence of Caries on Individual Tooth Surfaces and Its Distribution by Age and Gender in University Clinic Patients. Eur. J. Dent..

[26] Luan W., Baelum V., Fejerskov O., Chen X. (2000). Ten-Year Incidence of Dental Caries in Adult and Elderly Chinese. Caries Res..

[27] Hassan A., Khan J.A., Ali S.A. (2019). Caries Susceptibility of Proximal Surfaces in Permanent First Molars: A Cross Sectional Survey. J. Islam. Int. Med. Coll. JIIMC.

[28] Dosumu O.O., Ogunrinde J.T., Bamigboye S.A. (2014). Knowledge of Consequences of Missing Teeth in Patients Attending Prosthetic Clinic in U.C.H. Ibadan. Ann. Ib. Postgrad. Med..

[29] Scheiwiller M., Oeschger E.S., Gkantidis N. (2020). Third Molar Agenesis in Modern Humans with and without Agenesis of Other Teeth. PeerJ.

[30] Lindauer S.J., Laskin D.M., Tüfekçi E., Taylor R.S., Cushing B.J., Best A.M. (2007). Orthodontists’ and Surgeons’ Opinions on the Role of Third Molars as a Cause of DENTAL crowding. Am. J. Orthod. Dentofac. Orthop..

[31] Lyros I., Vasoglou G., Lykogeorgos T., Tsolakis I.A., Maroulakos M.P., Fora E., Tsolakis A.I. (2023). The Effect of Third Molars on the Mandibular Anterior Crowding Relapse—A Systematic Review. Dent. J..

[32] Assali A., Oualalou Y., Zaoui F. (2022). The Evolution of Third Molars in Orthodontics: What about Anterior Dental Crowding?—A Systematic Review. Integr. J. Med. Sci..

[33] León-López M., Cabanillas-Balsera D., Martín-González J., Montero-Miralles P., Saúco-Márquez J.J., Segura-Egea J.J. (2022). Prevalence of Root Canal Treatment Worldwide: A Systematic Review and Meta-Analysis. Int. Endod. J..

[34] American Association of Endodontists (2020). Glossary of Endodontic Terms.

[35] Özbaş H., Aşcı S., Aydın Y. (2011). Examination of the Prevalence of Periapical Lesions and Technical Quality of Endodontic Treatment in a Turkish Subpopulation. Oral Surg. Oral Med. Oral Pathol. Oral Radiol. Endod..

[36] Alnowailaty Y., Alghamdi F. (2022). Prevalence of Endodontically Treated Premolars and Molars With Untreated Canals and Their Association With Apical Periodontitis Using Cone-Beam Computed Tomography. Cureus.

[37] Orstavik D. (1996). Time-Course and Risk Analyses of the Development and Healing of Chronic Apical Periodontitis in Man. Int. Endod. J..

[38] Mosquera-Barreiro C., Ruíz-Piñón M., Sans F.A., Nagendrababu V., Vinothkumar T.S., Martín-González J., Martín-Biedma B., Castelo-Baz P. (2024). Predictors of Periapical Bone Healing Associated with Teeth Having Large Periapical Lesions Following Nonsurgical Root Canal Treatment or Retreatment: A Cone Beam Computed Tomography-Based Retrospective Study. Int. Endod. J..

[39] Daviet-Noual V., Ejeil A.-L., Gossiome C., Moreau N., Salmon B. (2017). Differentiating Early Stage Florid Osseous Dysplasia from Periapical Endodontic Lesions: A Radiological-Based Diagnostic Algorithm. BMC Oral Health.

[40] Mupparapu M., Shi K.J., Ko E. (2020). Differential Diagnosis of Periapical Radiopacities and Radiolucencies. Dent. Clin. N. Am..

[41] Ghafoor R. (2013). Conservative Management of Progressive External Inflammatory Root Resorption after Traumatic Tooth Intrusion. J. Conserv. Dent..

[42] Krupińska A.M., Bogucki Z. (2023). Evaluation of Patients’ Awareness and Knowledge Regarding Dental Implants among Patients of the Department of Prosthetic Dentistry at Wroclaw Medical University in Poland. Adv. Clin. Exp. Med..

[43] Elani H.W., Starr J.R., Da Silva J.D., Gallucci G.O. (2018). Trends in Dental Implant Use in the U.S., 1999–2016, and Projections to 2026. J. Dent. Res..

[44] Chen S.T., Buser D., Sculean A., Belser U.C. (2023). Complications and Treatment Errors in Implant Positioning in the Aesthetic Zone: Diagnosis and Possible Solutions. Periodontology 2000.

[45] Tikku A.P., Chandra A., Bharti R. (2010). Are Full Cast Crowns Mandatory after Endodontic Treatment in Posterior Teeth?. J. Conserv. Dent..

[46] White S.C., Pharoah M.J. (2009). Oral Radiology—Principles and Interpretation.

[47] Kamburoğlu K., Kolsuz E., Murat S., Yüksel S., Özen T. (2012). Proximal Caries Detection Accuracy Using Intraoral Bitewing Radiography, Extraoral Bitewing Radiography and PANORAMIC radiography. Dentomaxillofacial Radiol..

[48] Kweon H.H.-I., Lee J.-H., Youk T., Lee B.-A., Kim Y.-T. (2018). Panoramic Radiography Can Be an Effective Diagnostic Tool Adjunctive to Oral Examinations in the National Health Checkup Program. J. Periodontal Implant. Sci..

[49] Suomalainen A., Pakbaznejad Esmaeili E., Robinson S. (2015). Dentomaxillofacial Imaging with Panoramic Views and Cone Beam CT. Insights Imaging.

[50] Peretz B., Gotler M., Kaffe I. (2012). Common Errors in Digital Panoramic Radiographs of Patients with Mixed Dentition and Patients with Permanent Dentition. Int. J. Dent..

[51] Dziennik Ustaw Rzeczpospolitej Polskiej, Rozporządzenie Ministra Zdrowia z Dnia 8 Grudnia 2017 r. https://isap.sejm.gov.pl/isap.nsf/download.xsp/WDU20170002397/O/D20172397.pdf.

[52] Krois J., Ekert T., Meinhold L., Golla T., Kharbot B., Wittemeier A., Dörfer C., Schwendicke F. (2019). Deep Learning for the Radiographic Detection of Periodontal Bone Loss. Sci. Rep..

[53] Revilla-León M., Gómez-Polo M., Barmak A.B., Inam W., Kan J.Y.K., Kois J.C., Akal O. (2022). Artificial Intelligence Models for Diagnosing Gingivitis and Periodontal Disease: A Systematic Review. J. Prosthet. Dent..

[54] Revilla-León M., Gómez-Polo M., Vyas S., Barmak B.A., Galluci G.O., Att W., Krishnamurthy V.R. (2023). Artificial Intelligence Applications in Implant Dentistry: A Systematic Review. J. Prosthet. Dent..

